# Connective Tissue Growth Factor: From Molecular Understandings to Drug Discovery

**DOI:** 10.3389/fcell.2020.593269

**Published:** 2020-10-29

**Authors:** Zihao Chen, Ning Zhang, Hang Yin Chu, Yuanyuan Yu, Zong-Kang Zhang, Ge Zhang, Bao-Ting Zhang

**Affiliations:** ^1^School of Chinese Medicine, Faculty of Medicine, The Chinese University of Hong Kong, Hong Kong, China; ^2^Law Sau Fai Institute for Advancing Translational Medicine in Bone and Joint Diseases, School of Chinese Medicine, Hong Kong Baptist University, Hong Kong, China

**Keywords:** CTGF, fibrosis, aptamers, anti-CTGF, domain structure, CCN2

## Abstract

Connective tissue growth factor (CTGF) is a key signaling and regulatory molecule involved in different biological processes, such as cell proliferation, angiogenesis, and wound healing, as well as multiple pathologies, such as tumor development and tissue fibrosis. Although the underlying mechanisms of CTGF remain incompletely understood, a commonly accepted theory is that the interactions between different protein domains in CTGF and other various regulatory proteins and ligands contribute to its variety of functions. Here, we highlight the structure of each domain of CTGF and its biology functions in physiological conditions. We further summarized main diseases that are deeply influenced by CTGF domains and the potential targets of these diseases. Finally, we address the advantages and disadvantages of current drugs targeting CTGF and provide the perspective for the drug discovery of the next generation of CTGF inhibitors based on aptamers.

## Introduction

### Connective Tissue Growth Factor (CTGF) and CCN Family

CTGF, also known as CCN2, is a 38 kDa, cysteine-rich (22 cysteines in the N-terminal and 16 cysteines in the C-terminal region) (Boes et al., 1999), extracellular matrix protein that belongs to the CCN family of proteins (Abreu et al., 2002). The term ‘connective tissue growth factor’ was introduced to describe a novel polypeptide growth factor that stimulated DNA synthesis and chemotaxis in fibroblast (Bradham et al., 1991). There are other five CCN gene family genes: CCN1 (Cyr61), CCN3 (NOV), CCN4 (WISP1), CCN5 (WISP2), and CCN6 (WISP3) (Holbourn et al., 2008). The CCN acronym was introduced from the names of the first three members of the family to be discovered: Cyr61 (cysteine-rich protein 61), CTGF (connective tissue growth factor) and NOV (nephroblastoma overexpressed gene) (Bork, 1993). Expression of *CTGF* is crucial to embryonic development in childhood (Jun and Lau, 2011), for example, mice with *CTGF* knockout have multiple skeletal dysmorphisms and perinatal lethality (Lambi et al., 2012). Also, abnormal expression of *CTGF* was detected in several adulthood diseases including fibrosis and malignancy in major organs and tissues (Ramazani et al., 2018).

### Expression Profiles for CTGF in Human

**FIGURE 1:**
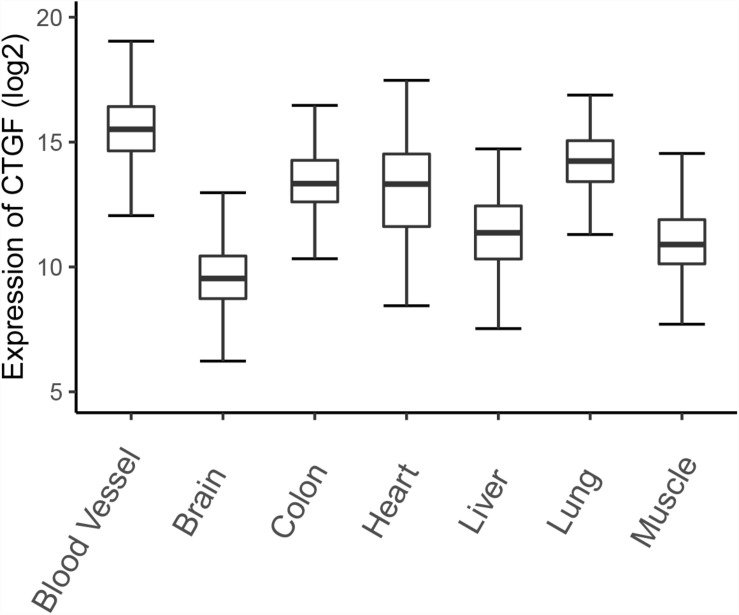
The expression of CTGF in different tissues. The expression data was downloaded from GTEx database and a total of 7313 samples (blood vessel: 1335; Brain: 2642; Colon: 779; Heart: 861; Kidney: 89; Liver: 226; Lung: 578; Muscle: 803) from normal human tissues were plotted.

Connective tissue growth factor expression was initially discovered in endothelial cells and fibroblasts associated with connective tissue regeneration and wound healing, and then was detected in many tissues (Bradham et al., 1991; Uhlen et al., 2015). Here, we illustrate the expression of *CTGF* in different organisms based on gene expression data from the Genotype-Tissue Expression (GTEx) project (Figure 1). The project contains expression data obtained from 54 non-diseased tissue sites across nearly 1000 individuals (Battle et al., 2017). *CTGF* expression is higher in blood vessels and lungs compared to other organs or tissues, which emphasize the role of CTGF in the development of blood vessels and lungs. Low expression of *CTGF* mRNA was observed in brain tissues by GTEx project, however, the previous study showed that the adult cerebral cortex strongly expresses *CTGF* mRNA (Heuer et al., 2003).

The proper expression of CTGF is essential for the physiological process of multiple organs such as bone, brain, heart, and lung. CTGF knockout mice demonstrated developmental skeletal malformations (Ivkovic et al., 2003). High expression of *CTGF* will negatively regulates myelination during development, which has been implicated in a range of neurodevelopmental disorders (Ercan et al., 2017). *CTGF* mRNA was highly expressed in developing blood vessels and large blood vessels of the adult heart, suggesting that it may be involved in the maintenance of blood vessel integrity during adulthood (De Sousa Lopes et al., 2004). The absence of *CTGF* and/or its protein product, CTGF, may induce pulmonary hypoplasia by disrupting basic lung developmental processes (Baguma-Nibasheka and Kablar, 2008).

### Protein Domains in CTGF

**FIGURE 2:**
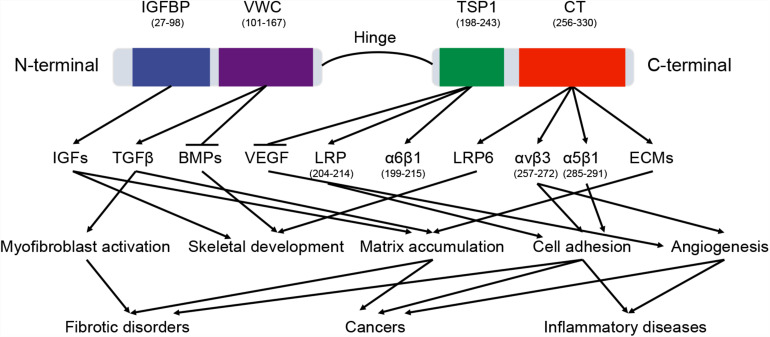
The domains of CTGF protein. CTGF domains interact with a variety of molecules, including cytokines, growth factors, receptors, and matrix proteins. These interactions regulate multiple signaling pathways in physiological and pathological processes. The arrow and horizontal line correspond to promotion and counteraction, respectively.

*CTGF* (6q23.2) is a relatively short gene and consists of 5 exons that code for a 349-amino acid protein, the first exon codes for a signal peptide (for secretion) and exons 2–5 code for each of the four different domains (Arnott et al., 2011). The four functional domains are insulin-like growth factor binding protein (IGFBP), von Willebrand factor type C repeat (VWC), thrombospondin type-1 repeat (TSP1 or TSR), and cysteine knot-containing domain (CT) (Figure 2). IGFBP and VWC domains constitute the N-terminal half of CTGF which is separated from the C-terminal half that contains TSP1 and CT domains by a ‘hinge’ region (Anna et al., 2015). In this study, the boundaries for domains were defined by ‘P29279’ of UniProtKB database with IGFBP domain (GLN27-LYS98), VWC domain (ALA101-ASP167), TSP1 domain (ASN198-GLU243), and CT domain (CYS256-PRO330).

The functions of CTGF domains are different because of their distinct bindings with specific proteins in various signaling pathways (Figure 2). Since these binding proteins participate in a number of physiological processes, CTGF has been shown to regulate a wide range of important functional pathways, including adhesion, mitogenesis, and chemotaxis, cell survival, differentiation, angiogenesis, chondrogenesis, tumorigenesis, and wound healing (Holbourn et al., 2008). Although some biological functions are directly related to an individual functional domain, many functions are demonstrated to be the consequence of domains acting in concert. For example, truncated CTGF domains (N-terminal fragment and C-terminal fragment), can function independently to stimulate differentiation or proliferation of fibroblast and to increase collagen synthesis (Gary and Matthew, 2005).

Connective tissue growth factor protein contains 38 fully conserved cysteine residues, which are evenly spread throughout the protein (1 in signal peptide region, 11 in IGFBP, 10 in VWC, 6 in TSP1, and 10 in CT) except for a cysteine free-region between Asp167 and Asn198 (‘hinge region’) (Brigstock, 1999). Disulfide bond was formed by a reaction between the sulfhydryl side chains of two cysteine residues, and had an essential role in the stabilization of peptide and protein structures and modulation of biological activities (Wiedemann et al., 2020). In CTGF, the functions of disulfide bond can be summarized: (1) To stabilize the structure of CTGF by linking the secondary structures such as β-sheets (Holbourn et al., 2008). (2) To form the ‘cysteine knot’ in CT domain of CTGF, and the cysteine knot is crucial for dimerization of proteins and binding with other receptors or growth factors (Holbourn et al., 2008).

## The Structure and Biological Functions of CTGF Domains

Three-dimensional (3D) structure of the protein is crucial for its interaction with other molecules and biological functions. Currently, there is no accurate structural information about the CTGF. Homology modeling is a computational method and predicts reliable 3D structure of a query protein through the sequence alignment of template proteins (Meier and Soding, 2015). In current study, we predicted 3D structures of individual domains of CTGF by using Modeller (Webb and Sali, 2016). The amino acid sequence of CTGF was retrieved from NCBI and the accession number ‘CAG46534.1’ was selected for the present study. The templates for predicting the structure of domains were searched by Swiss-model server and then listed in Table 1 (Waterhouse et al., 2018). For each domain, 50 structures were created by the Modeller and the predicted structure with lowest dope value was considered the highest quality and then selected for structure evaluation (Supplementary Figures S1A–D). The structure evaluation was done by the Ramchandran plot in PROCHECK, and the results showed a good quality of structure since all residues were located in the allowed area (Table 2).

**TABLE 1:** Summary of the templates for the prediction of CTGF domains.

Template	Protein	Targeting domain	Coverage	Sequence similarity
3TJQ	HtrA1	IGFBP	0.76	0.40
1WQJ	IGFBP4	IGFBP	0.79	0.34
3ZXB	SIBD-1	IGFBP	0.81	0.34
5NB8	CCN3	VWC	0.99	0.52
1U5M	Collagen IIA	VWC	0.82	0.33
5NIR	Collagen 2A	VWC	0.82	0.33
6RK1	CCN3	TSP1	0.98	0.57
3GHN	ADAMTS13	TSP1	0.91	0.38
3T5O	CC6	TSP1	0.89	0.39
2K8P	Sclerostin	CT	0.80	0.34
4NT5	CTCK	CT	0.84	0.34
4X1J	NBL1	CT	0.81	0.32

**TABLE 2:** Ramachandran plot analysis.

Ramachandran Plot Calculation	IGFBP %	VWC %	TSP1 %	CT %	FL-CTGF %
**Residues in most favored regions**	81.5	96.2	87.8	75.0	80.2
**Residues in additional allowed regions**	13.0	3.8	9.8	20.3	16.2
**Residues in generously allowed regions**	5.6	0.0	2.4	4.7	3.6
**Residues in disallowed regions**	0.0	0.0	0.0	0.0	0.0

### IGFBP Domain

**FIGURE 3:**
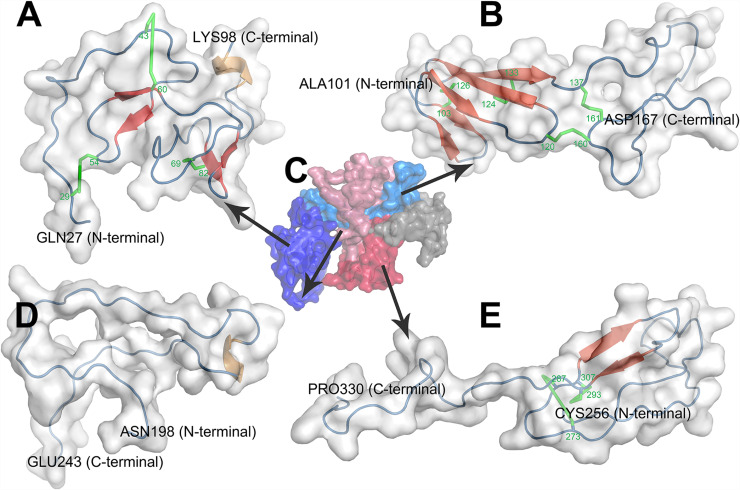
Homology modeling of structures of CTGF domains and full length CTGF using MODELLER. **(A)** IGFBP domain; **(B)** VWC domain; **(C)** full length CTGF; **(D)** TSP1 domain; **(E)** CT domain. **(C)** Domains were represented by different colors (IGFBP domain: deep blue; VWC domain: light blue; linkage region: gray; TSP1 domain: light red; CT domain deep red). In the structure of each domain, secondary structures were represented by colors (β-sheets: red; coil: blue; α helix: brown; disulfide bond: green).

The first domain of CTGF, named as IGFBP domain, is coded by the exon 2 of *CTGF* and located in the sequence of Gln27-Lys98. The name of IGFBP domain comes from its similar structure with IGFBP family proteins which contain a conserved motif ‘GCGCCXXC,’ thus CTGF has been characterized as a member of the IGFBP superfamily (Krupska et al., 2015). There are two major sub-domains in the CTGF-IGFBP domain (Figure 3A), and the first sub-domain (SD1) is constituted by a 2-stranded β-sheet and two disulfide bonds in the N-terminal of IGFBP domain. The β-sheet, disulfide bond, and conserved ‘GCGCCxxC’ motif, forms a rigid base that supports the binding of CTGF and IGF (Sitar et al., 2006). The second sub-domain (SD2) consists of a globular sub-domain centered around a 2-stranded anti-parallel β-sheet in the C-terminal of IGFBP domain. The site for binding insulin-like growth factors (IGFs) is located in the SD2 (Sitar et al., 2006).

IGFBP family proteins exert critical roles in a variety of cellular functions including amino acid and glucose uptake, cell cycle, cell proliferation, cell differentiation, and immune response through its binding with IGFs (Jones and Clemmons, 1995). As a member of IGFBP family proteins, the specificity of binding between IGFs and CTGF is further confirmed by competitive affinity binding assays using unlabeled IGF-I and IGF-II (Kim et al., 1997). However, the affinity of CTGF-IGFs interaction is only about one percent of the affinity of other IGFBP family proteins (IGFBPI-VI) to IGFs (Yang et al., 1998). The lower affinity of CTGF with IGFs which is thought to be the consequence of the lack of C-terminal of IGFBP family proteins (Kim et al., 1997). This low affinity makes CTGF promote IGFs’ function by prolonging the circulating half-life of IGFs and increasing the bioavailability of IGFs (Lam et al., 2003), unlike other classic IGFBPs which block the interaction between IGFs and their receptors due to their high affinity to IGFs (Wang et al., 2001; Allard and Duan, 2018). For example, overexpression of CTGF enhances the expression of IGFs, activates IGFs mediated proliferation and proteoglycan synthesis in cultured chondrocytes (Tomita et al., 2013). CTGF is also found to bind with IGFs and contributes to the matrix accumulation in tubulointerstitial fibrosis (Lam et al., 2003). Apart from IGF-dependent manner, IGFBP domain enables the linkage of CTGF to fibronectin, a prominent component of extracellular matrix (ECM), and enhances cell adhesion and matrix deposition which leads to liver fibrosis (Pi et al., 2008). Taken together, the main functions of IGFBP domain of CTGF include: (a) To stimulate proliferation of chondrocytes; (b) To increase matrix accumulation in both IGF-dependent and IGF-independent manner.

### VWC Domain

The second domain presented in the N-terminal of CTGF is VWC domain which is coded by the exon 3 of *CTGF* and located in Ala101-Asp167. This domain, also known as the chordin-like cysteine rich (CR) repeat which has been shown to bind members of the transforming growth factor-β (TGFβ) superfamily, is found in more than 500 ECM proteins (Zhang et al., 2007). There are two sub-domains in the VWC domain of CTGF (Figure 3B). The first sub-domain (SD1) is more structured and consists of a short 2-stranded anti-parallel β-sheet followed by a 3-stranded anti-parallel β-sheet in the N-terminal part of VWC domain. The 3-stranded anti-parallel β-sheet is supported by a disulfide bond between its second and third strand, and another disulfide bond forms between short 2- stranded β-sheet and 3-stranded anti-parallel β-sheet. The second sub-domain (SD2) is comprised of random coils with no secondary structure elements in the C-terminal part of VWC domain. SD2 is devoid of regular secondary structure, although it is tethered by one disulfide bond within itself and one disulfide bond to SD1 (O’Leary et al., 2004; Xu et al., 2017).

The cellular function of VWC domain may come from the interaction with TGFβ and bone morphogenetic proteins (BMPs) (Abreu et al., 2002). CTGF binds TGFβ with low affinity and functions as a chaperone to escort TGFβ to its receptors (Abreu et al., 2002). CTGF can strengthen the profibrotic functions of TGFβ in particular proliferation of fibroblasts and secretion of ECM proteins by the fibroblasts. The strengthened TGFβ induces the higher expression of CTGF, which forms a positive feedback loop (Abreu et al., 2002). This partnership has been implicated in many diseases (e.g., cancers and fibrotic diseases) (Brigstock, 2010). Besides, CTGF acts via glial-derived TGFβ, whose activity it potentiates, promoting SMAD-dependent apoptosis of newborn neurons in the glomerular layer of brain (Khodosevich et al., 2013). VWC domain also exists in crossveinless-2 (CV2), and the similarity of VWC domain in CTGF to CV2 is 31.8%. The SD1 of VWC domain of CV2 is responsible for CV2-BMP2 binding (Zhang et al., 2008), implying CTGF might also bind to BMP2 by SD1 of VWC domain. Binding of CTGF-VWC domain to BMPs inhibits BMPs signaling by preventing the binding of BMPs to their cognate receptors (Abreu et al., 2002). To date, BMP family proteins, such as BMP-2 (Mundy et al., 2014), BMP-4 (Abreu et al., 2002), BMP-6 (Falke et al., 2016), and BMP-7 (Nguyen et al., 2008) have been demonstrated to be negatively influenced by CTGF in different cell types. The previous study found that CTGF knockout increased BMP signaling and overexpression of CTGF decreased BMP signaling in osteoblasts (Mundy et al., 2014).

Taken together, the major functions of VWC domain of CTGF may include: (a) To interact with TGFβ to induce fibrosis and apoptosis of newborn neurons; (b) To bind with BMPs to suppress the differentiation of chondrocytes and osteoblasts.

The exon 3 of *CTGF* not only encodes the VWC domain but also a region devoid of cysteines that may serve as a ‘hinge’ connecting the N-terminal and C-terminal halves of CTGF. This ‘hinge’ region is unique to each CCN family member and contains an abundance of charged amino acids. Matrix metalloproteinases (MMPs) have been shown to cleave CTGF in the unstructured hinge region between VWC domains and TSP1 domain (Hashimoto et al., 2002). The cleavage of hinge is very crucial for CTGF’s function. Since the N-terminal fragment conceals the binding site of C-terminal fragment of CTGF in intact CTGF, the full length of CTGF is an inactive precursor (Kaasboll et al., 2018). The cleavage of the CTGF hinge by MMPs releases the C-terminal fragment which is responsible for activation of Akt and the ERK pathway (Kaasboll et al., 2018).

### TSP1 Domain

The third domain of CTGF, also known as TSP1 domain, corresponds to the sequence of Asn198-Glu243. TSP1 domain was firstly identified in human endothelial cell thrombospondin-1 protein and then turned out to be one of the most common motifs in a variety of extracellular proteins (Lawler and Hynes, 1986). CTGF-TSP1 domain contains six cysteines and an identical motif ‘CSXXCG’ to TSP1 domain of thrombospondin-1 protein (Tucker, 2004). The main character of TSP1 domain of thrombospondin-1 protein is the three anti-parallel strands, placing the N- and C-termini at opposite ends of domain. And strand I is more irregular since it does not form a regular β strand, while strands II and III form a regular antiparallel-sheet (Tan et al., 2002). However, in the structure of CTGF-TSP1 domain, none of the strand I (Cys199-Gly214), strands II (Ser218-Asn223) and strands III (Lys232-Cys237) form a regular β-sheet (Figure 3D). CTGF-TSP1 domain is more open, with strand I, strand II and strand III are more separate from each other, which makes it less possible to form H-bonds, disulfide bonds, and β-sheets.

The main binding proteins of TSP1 domain include integrin α6β1, low density lipoprotein receptor-associated protein (LRP), vascular endothelial growth factor (VEGF). CTGF could bind with integrin α6β1 by ‘CLVQTTEWSACSKTCGM’ (Cys199-Met215) in TSP1 domain, evidenced by the inhibited collagen deposition in fibroblasts caused by exogenous CTGF peptide (Cys199-Met215) treatments (Heng et al., 2006). In addition, CTGF could bind with LRP by ‘TEWSACSKTCG’ (Thr204-Gly214) in TSP1 domain, evidenced by a 50% decrease in hepatic stellate cell adhesion by exogenous CTGF peptide (Thr204-Gly214) treatments (Gao and Brigstock, 2003). However, the accurate binding site of TSP1 domain to VEGF remains unknown.

Integrin α6β1 is a well characterized laminin receptor and participates in ECM interactions (Koivisto et al., 2014). CTGF mediates collagen deposition by the binding between TSP1 domain and integrin α6β1 (Heng et al., 2006). LRP acts as a signaling receptor and can regulate diverse processes such as repair, remodeling, and embryonic development (Gao and Brigstock, 2003). Hepatic stellate cells (HSCs) adhesion, which is a critical event in hepatic fibrosis (March et al., 2007), is promoted by CTGF through its binding to LRP in CTGF-TSP1 domain (Gao and Brigstock, 2003). VEGF is a strong angiogenic mitogen and plays an important role in angiogenesis under various pathophysiological conditions (Inoki et al., 2002). CTGF inhibits the function of VEGF through the binding between TSP1 domain and VEGF, which leads to the decreased angiogenic activity (Inoki et al., 2002). The main function of TSP1 domain can be concluded: (a) To promote collagen deposition by integrin α6β1; (b) To increase HSCs adhesion by LRP; (c) To inhibit angiogenesis by VEGF.

### CT Domain

The fourth domain which is located in Cys256-Pro330 and encoded by exon 5 of *CTGF*. This domain contains a ‘cystine knot’ (CT) structure, therefore this domain is named as CT domain. The CT domain is also presented in growth factors including the TGFβ superfamily, platelet derived growth factor (PDGF) and nerve growth factors (NGFs) (Schlunegger and Grutter, 1993). Cysteine knot is an 8-residues ring based around a two-stranded anti-parallel β-sheet (with each strand at least 4 residues long) linked by disulfide bonds (Molesini et al., 2017). In the structure of CTGF-CT domain, cysteine knot consists a two-stranded anti-parallel β-sheet and two disulfide bonds (Figure 3E). The CT is highly conducive to protein stability which can be attributed to conformational rigidity endowed by disulfide bonds of the CT (Sherbet, 2011). The CT also probably has a role in dimerization of proteins and may be involved in receptor binding of growth factors (Sherbet, 2011).

Cystine knot domain has been reported to bind with ECM proteins (e.g., fibronectin), integrins (e.g., α5β1 and αvβ3) and LRP6. CT domain enables the linkage of CTGF to fibronectin, and promotes cell adhesion and migration (Pi et al., 2008). Integrin α5β1 is involved in the interaction of CT domain with the fibronectin which contributes to the ECM accumulation in fibrosis disorders (Gressner and Gressner, 2008). CTGF enhances integrin-fibronectin binding and their function by forming a ternary complex (Hoshijima et al., 2006). The sequence ‘GVCTDGR’ (Gly285-Arg291) mediates the binding between CT and integrin α5β1 and promotes cell adhesion and migration. Integrin αvβ3 plays important roles in regulating cell adhesion and migration, as well as angiogenesis (Babic et al., 1999). CTGF-CT domain can bind with αvβ3 and promote the above functions of αvβ3 in the sequence of ‘IRTPKISKPIKFELSG’ (Ile257–Gly272) (Gao and Brigstock, 2004). LRP6 is a key protein in Wnt pathway and contributes to pericyte migration, myofibroblast differentiation, and matrix accumulation (Ren et al., 2013). It has been widely reported that CTGF can bind with LRP6 and further regulates Wnt signaling (Rooney et al., 2011; Yang et al., 2015; Johnson et al., 2017). However, the actual role of CTGF-LRP6 interaction in regulating Wnt signaling is still unclear. Most studies showed that CTGF could activate Wnt signaling (Rooney et al., 2011; Yang et al., 2015; Johnson et al., 2017). Whereas it also has been reported that CTGF competes with Wnt family members for binding to LRP6 in Xenopus laevis embryos (Mercurio et al., 2004). The main function of CT domain includes: (a) To induce ECM deposition by fibronectin; (b) To promote cell adhesion and angiogenesis by integrins α5β1 and αvβ3, respectively; (c) To regulate Wnt signaling by LRP6.

### Full Length CTGF

Homology modeling of full length CTGF (FL-CTGF) could not be performed due to the lack of acceptable templates for FL-CTGF. Thus, a strategy of predicting the structure of FL-CTGF by assembling the structures of domains (Ganugapati and Akash, 2017) was adopted in this research. The 3D structure files of these four domains and the alignment files were used as templates for Homology modeling to predict the 3D structure of FL-CTGF. The predicted structure with the lowest dope value was chosen for the best structure of FL-CTGF, and the structure assessment by Ramachandran plot indicated the good quality of FL-CTGF structure since none of the residues were located in disallowed regions (Table 2). We calculated the root-mean-square deviation (RMSD) (Maiorov and Crippen, 1994) to measure the similarity between structures of individual domain and FL-CTGF, and the results showed that the FL-CTGF retained the same architecture as of individual domain since zero RMSD were found. We also found that there were some interactions between domains. In this study, a pair of residues were defined to be in contact when the distance between their C-beta atoms (C-alpha in the case of glycine) was less than 8 Angstroms (Adhikari and Cheng, 2017). The result indicated 60 pairs of interacted residues between VWC and TSP1 domain, and 31 pairs of interacted residues between VWC and CT domain (Supplementary Table S1).

## Molecular Understandings of CTGF in Diseases

As introduced above, CTGF has important roles in many biological processes, including cell adhesion, migration, proliferation, angiogenesis, skeletal development, and tissue wound repair. Meanwhile, it is critically involved in various diseases, including cancers, fibrotic diseases, and inflammatory diseases.

### Cancers

Key roles for CTGF are to promote myofibroblast differentiation and angiogenesis. Similar mechanisms are active in different cancers where CTGF is expressed. To date, 30 types of human cancers have been linked to deregulated aberrant expression of CTGF (Table 3). Cancers can be divided into 3 categories according to the role of CTGF in tumor development: tumor promotion (Group I), suppression (Group II), and both (Group III).

**TABLE 3:** Summary of the relationship between CTGF expression and tumor progression.

Cancer type	Correlation with tumor progression.
**Group I**	**Tumor promoter**
**Acute lymphoblastic leukemia**	Higher CTGF expression corresponds to the worsening of overall survival (Sala-Torra et al., 2007).
**Glioma**	Positive correlations exist between *CTGF* mRNA levels versus tumor grade, gender, and pathology (Xie et al., 2004).
**Breast cancer**	Elevated levels of CTGF in primary breast cancers are associated with more advanced features (Xie et al., 2001).
**Gastric cancer**	Patients with elevated CTGF expression have more lymph node metastases and shorter survival time (Liu et al., 2008).
**Hepatocellular carcinoma**	The expression of CTGF is associated with poor survival (Xiang et al., 2012).
**Ileal carcinoid**	CTGF in carcinoid tumors is significantly increased versus normal carcinoids (Jacobson and Cunningham, 2012).
**Melanoma**	CTGF is overexpressed in malignant melanoma and promotes cell invasion and migration (Braig et al., 2011).
**Mesothelioma**	Elevated expression of CTGF promotes mesothelioma growth (Ohara et al., 2018).
**Esophageal cancer**	Forced expression of CTGF significantly increases tumor formation (Deng et al., 2007).
**Pancreatic cancer**	CTGF antibody therapy inhibits pancreatic tumor growth and metastasis (Dornhofer et al., 2006).
**Prostate cancer**	CTGF promotes prostate carcinoma to metastasize in the bone (Zhang et al., 2018).
**Thyroid cancer**	CTGF is overexpressed in papillary thyroid carcinoma and promotes the growth of papillary thyroid cancer cells (Cui et al., 2011).
**Head and neck squamous cell carcinoma (HNSCC)**	Up-regulation of CTGF is observed in tumor specimens from patients with HNSCC (Mullis et al., 2008).
**Cervical cancer**	CTGF is upregulated in late stage cancer compared to early stage cancer (Wong et al., 2006).
**Rhabdomyosarcoma**	Inhibition of CTGF induces rhabdomyosarcoma cell death and decrease tumor angiogenesis (Croci et al., 2004).
**Myofibroblastic tumor**	Myofibroblastic tumor expresses CTGF in both endothelial cells and vimentin-positive tumor cells, particularly those around the blood vessels (Kasaragod et al., 2001).
**Renal cell carcinoma**	CTGF is found to be over-expressed in the renal cell carcinoma tissues (Chintalapudi et al., 2008).
**Endometrial cancer**	The expression of CTGF is significantly higher in endometrial cancers compared to normal tissues (Li et al., 2019).
**Bladder cancer**	Down-regulation of CTGF suppresses proliferation, migration, and invasion of bladder cancer cells *in vitro* and targeting of CTGF decelerated xenograft growth *in vivo* (Wang X. et al., 2017).
**Osteosarcoma**	CTGF induces osteosarcoma metastasis via the αvβ3 integrin/FAK/PI3K/Akt/NF-κB signaling pathway (Hou et al., 2018).
**Group II**	**Tumor suppressor**
**Colorectal cancer**	Patients with low CTGF expression have shorter survival time (Lin et al., 2005).
**Lung cancer**	Low CTGF levels correlate with high tumor stage and metastasis (Chen et al., 2007).
**Oral squamous cell carcinoma**	CTGF suppresses tumor cell growth in a human oral squamous cell carcinoma-derived cell line (Moritani et al., 2003).
**Meningioma**	*CTGF* mRNA levels are lower in recurrences compared to the primary tumor (Perez-Magan et al., 2010).
**Chondrosarcoma**	CTGF expression is negatively correlated with proliferation and tumor grade of chondrosarcoma (Shakunaga et al., 2000).
**Intrahepatic cholangiocarcinoma**	Low CTGF expression predicts the recurrence of intrahepatic cholangiocarcinoma (Gardini et al., 2005).
**Nasopharyngeal carcinoma (NPC)**	Reduced expression of CTGF promotes cell proliferation, migration, invasion in NPC (Zhen et al., 2014).
**Group III**	**Complex correlation**
**Ovarian cancer**	*CTGF* mRNA is reduced in ovarian cancer cell lines compared with the normal cells. However, CTGF expression was higher in the advanced stages of ovarian cancer (Kikuchi et al., 2007).
**Wilms tumor**	CTGF is activated in early tumorigenesis, while its expression decreases with tumor progression (Zirn et al., 2006).
**Gallbladder cancer**	CTGF is found to be overexpressed in primary gallbladder cancer, compared with non-neoplastic gallbladder epithelium. But gallbladder cancer with high CTGF expression has a favorable survival (Alvarez et al., 2008).

In Group I, CTGF expression is positively associated with poor survival outcomes and clinicopathological findings, including advanced stage, larger tumor size, and increased metastasis. Epithelial to mesenchymal transition (EMT) is the process where epithelial cells acquire a mesenchymal cell phenotype which is more prone to migrate and invade (Tsai and Yang, 2013). CTGF and its binding proteins TGFβ are major initiators of EMT process and contribute to the tumor progression in tumors such as breast cancer (Wendt et al., 2010; Zhu et al., 2015). Another key role of CTGF in tumor development is angiogenesis. The high expression level of CTGF enhances angiogenesis in breast cancer and osteosarcoma by integrin αvβ3 (Shimo et al., 2001; Wang L. H. et al., 2017). Integrin αvβ3 plays a role in angiogenesis mostly by sprouting endothelial cells, therefore promotes invasion and metastasis in cancers such as glioma and breast cancer (Lorger et al., 2009). Taken together, CTGF interacts with TGFβ and integrin αvβ3, respectively, and contributes to cancer development in Group I. The binding domain of CTGF for TGFβ is VWC domain, and CT domain is responsible for the binding of CTGF to integrin αvβ3.

In Group II, CTGF may act as a tumor suppressor. In these cases, the expression level of CTGF is usually lower in the tumor tissues compared with adjacent normal tissues. CTGF expression is suppressed in non-small cell lung cancer cells, and the decreased expression of CTGF may play a role in lung tumorigenesis by allowing IGF-I to have greater progrowth activity (Chien et al., 2006), which is different from the pro-IGF function in physiological endochondral ossification process. In addition, CTGF can inhibit lung adenocarcinoma growth *in vitro* and *in vivo* by reducing VEGF gene expression and its subsequent angiogenic effects (Chang et al., 2006). Depressed CTGF triggers non-canonical Wnt pathway-mediated intestinal cancer progression through an increase in cancer stemness and acquisition of chemoresistance (Lin et al., 2005; Kim et al., 2020). Taken together, IGFBP, TSP1, and CT domains of CTGF are responsible for interaction with IGF-I, VEGF, and LRP6, respectively, to regulate the cancer progression in Group II.

In Group III, CTGF can act both positively and negatively in tumorigenesis and tumor progression. CTGF expression is lower in ovarian cancer cell lines compared to normal ovarian cells, whereas CTGF expression has a higher expression in advanced stages (stages III and IV) than earlier stages (stages I and II) (Kikuchi et al., 2007). The expression of CTGF is higher in primary gallbladder cancer compared with normal gallbladder tissue, however, CTGF expression in advanced gallbladder cancer is reduced to the levels of normal gallbladder epithelium. High CTGF expression correlates with a better survival outcome in advanced gallbladder cancer (Alvarez et al., 2008). To date, the detailed functions and mechanisms of CTGF in Group III cancers remain unclear.

Therefore, whether CTGF has a positive or negative correlation with tumor growth is related to the type and stage of cancer. The bindings between proteins with CTGF domains which exert distinct effects on tumor development may lead to the different functions of CTGF in cancers (Jun and Lau, 2011). For example, CTGF expression can inhibit tumor growth by reducing VEGF mediated angiogenic effects through TSP1 domain (Inoki et al., 2002; Chang et al., 2006) and enhance angiogenesis and osteosarcoma through the binding between CT domain and integrin αvβ3 (Shimo et al., 2001; Wang L. H. et al., 2017). However, current investigations for CTGF in cancers have been conducted in different subjects including cell lines, animals, and patients, which may cause inconsistent conclusions. Moreover, some limitations such as small sample size might compromise the results. Therefore, more investigations for the functional roles and underlying mechanisms of CTGF in different cancers are required.

Despite CTGF shows different functions in tumorigenesis, targeting CTGF has shown therapeutic promises in specific cancers such as breast cancer and pancreatic cancer (Jun and Lau, 2011). In breast cancer, blocking CTGF by its VWC domain greatly decreased osteolytic bone metastasis and angiogenesis (Shimo et al., 2006). In pancreatic cancer, tumor cell growth can be dramatically reduced by using genetic inhibition of CTGF (Bennewith et al., 2009). For these cancers, the VWC domain and CT domain of CTGF has a critical role in promoting tumor progression, which could be the potential targets.

### Fibrotic Disorders

Fibrosis is defined by the pathological accumulation of ECM proteins, which is in essence an exaggerated wound healing response that interferes with normal organ function (Neary et al., 2015). Fibrosis contributes to the morbidity and mortality associated with organ failure in a variety of chronic diseases affecting the lung, kidneys, eyes, heart, liver, and skin (Wang X. et al., 2011). High expression of CTGF is induced by many cytokines (e.g., TGFβ, VEGF, and integrins) and conditions associated with pathophysiology in fibrotic tissue (Oliver et al., 2010). CTGF activates myofibroblast formation by transdifferentiating other cells, such as resident fibroblasts, and epithelial cells (Lipson et al., 2012). CTGF also increases the expression of different cytokines, including TGFβ, VEGF, and integrins, which in turn further increase CTGF expression, resulting in positive feedback loops (Yang et al., 2010). Among various fibrotic diseases, CTGF has been extensively studied in pulmonary fibrosis (Allen and Spiteri, 2002), Cardiac fibrosis (Dorn et al., 2018), liver fibrosis (Gressner and Gressner, 2008), renal fibrosis (Toda et al., 2018), Duchenne muscular dystrophy (DMD) (Morales et al., 2011), and ocular disorders (Kubota and Takigawa, 2015).

Idiopathic pulmonary fibrosis (IPF) is a chronic, progressive, and fatal pulmonary fibrotic disease with unknown etiology (Puglisi et al., 2016). Excessive ECM disposition and fibrosis due to the imbalance between the profibrotic and antifibrotic events is the primary event of IPF. Plasma CTGF levels are significantly higher in patients with IPF than healthy volunteers (Kono et al., 2011). CTGF-deficient animals have fewer myofibroblasts and ECM disposition, indicating that CTGF is necessary for the induction of fibrosis in these animals (Liu et al., 2011). Overexpression of CTGF, in cooperation with TGFβ, is profibrotic and exacerbates ECM production in animal lung tissue (Sonnylal et al., 2010). The severity of alveolitis and fibrosis in the mouse model of lung fibrosis was markedly attenuated by CTGF inhibition (Wang X. et al., 2011).

COVID-19 (Coronavirus disease 2019) is caused by a novel coronavirus, named as severe acute respiratory syndrome coronavirus 2 (SARS-CoV-2), and has emerged as a pandemic and a public health crisis of global proportions (Harapan et al., 2020). The pathological changes of severe COVID-19 include diffuse alveolar damage and multi-organ dysfunction (Li et al., 2020). Lung fibrosis is deeply involved in COVID-19 development. Pulmonary fibrosis was observed in patients with COVID-19 (Ye et al., 2020) and in the fatal cases of COVID-19 (Delpino and Quarleri, 2020; George et al., 2020). About 40% of patients with COVID-19 develop acute respiratory distress syndrome (ARDS) that results in lung fibrosis as a long-term outcome (Gibson et al., 2020). *CTGF* and *TGFβ* were found to be increased in the alveolar epithelial cells inoculated with SARS-CoV-2 (Xu et al., 2020). As a potential therapeutic target of fibrosis, the anti-CTGF therapies are desirable to mitigate lung fibrosis in severe COVID-19 and facilitate COVID-19 recovery.

Chronic liver disease can lead to the permanent loss of hepatocyte mass and replacement by fibrotic tissue, which can ultimately lead to severe architectural disturbance (Ramazani et al., 2018). CTGF is highly expressed in fibrotic livers and its level in sera correlates significantly with fibrogenic activity (Gressner and Gressner, 2008). Although a lot of attention has gone to the collaboration of TGFβ and CTGF to pro-fibrotic effect, CTGF may also contribute to the ECM accumulation in fibrotic tissues by direct molecular interactions with matrix components (Gressner and Gressner, 2008). For example, CTGF induces adhesion of activated HSCs by the interaction between CT domain to integrin αvβ3 and heparan sulfate proteoglycans (HSPGs) (Gao and Brigstock, 2004). Some studies have shown that CTGF inhibition could reduce liver fibrosis in experimental and clinical settings (Han et al., 2012; Schippers et al., 2017).

Cardiac fibrosis is a common pathologic consequence of stress insult to the heart and often occurs in the context of hypertension and diabetes mellitus. Cardiac fibrosis is characterized by abnormal deposition of fibrotic ECM that compromises cardiac function (Dorn et al., 2018). The abundance of mRNA for *CTGF* is positively correlated with myocardial fibrosis areas in diastolic dysfunction patients (Koitabashi et al., 2007). Serum response factor (SRF), a transcription factor that could promote the proliferation of cardiac fibroblasts, induced the expression of CTGF in mouse cardiomyocytes (Angelini et al., 2015). CTGF deletion effectively inhibited TGFβ-induced collagen production in mouse embryonic fibroblasts (Dorn et al., 2018). Administration of the CTGF-antibody significantly improved cardiac fibrosis and enhanced left ventricular function in the mouse model (Koshman et al., 2015). However, CTGF inhibition for the treatment of heart diseases has not been studied in clinical trials.

Kidney fibrosis is a final common pathway of chronic kidney disease (CKD) irrespective of etiology. The excessive deposition of ECM in the interstitial compartment leads to scar tissue formation. Upon injury, tubule epithelial cells may undergo EMT which results in tubular function impairment, triggers cell cycle arrest and promotes the release of CTGF (Zhou and Liu, 2016). Thus, overexpression of CTGF has been observed in many renal fibrotic disorders including diabetic nephropathy (DN), chronic allograft nephropathy, IgA nephropathy (Yin and Liu, 2019). CTGF derived from proximal tubule epithelial cells (PTC) mediated both renal fibroblast proliferation and myofibroblast differentiation. Moreover, it was shown that CTGF expression was induced in primary PTC, through a myocardin-related transcription factor (MRTF) and SRF pathway (Sakai et al., 2017). Apart from TGFβ and integrins, CTGF could also interact with LRP6 and activate Wnt pathway to enhance collagen expression in tubular epithelial cells *in vitro* (Yang et al., 2015). CTGF inhibition significantly ameliorates the development of renal interstitial fibrosis *in vivo* (Yokoi et al., 2004).

In DMD and the mdx mouse model, the resultant myofibre degeneration and necrosis caused by the absence of the cytoskeletal protein dystrophin lead to a progressive loss of muscle mass, increased fibrosis and ultimately fatal weakness (Morales et al., 2013). Elevated levels of *CTGF* mRNA is found by RT-PCR analysis in the muscles of DMD patients (Sun et al., 2008). And CTGF activity in the DMD model is positively correlated with the number of necrotic-regenerative foci and mRNA levels of fibrotic markers (Morales et al., 2018). TGFβ inhibition or CTGF suppression can reduce the fibrotic phenotype in the mdx mouse model (Taniguti et al., 2011; Morales et al., 2013). CTGF causes accumulation of ECM through ERK phosphorylation possibly via the interaction with HSPGs (Ramazani et al., 2018). Anti-CTGF treatment shows better muscle strength in isolated muscles and reduced skeletal muscle impairment, apoptotic damage, and fibrosis (Morales et al., 2013).

A number of ocular diseases such as diabetic retinopathy, myopia, and glaucoma are shown to be associated with aberrant CTGF expression (Winkler et al., 2012; Yan and Chaqour, 2013). In diabetic retinopathy, increased CTGF contributes to the increased levels of TGFβ, retinal cell apoptosis, and the number of myofibroblasts (Yang et al., 2010). Synergistically with TGFβ, CTGF causes cellular changes (myofibroblastic phenotype) and ECM accumulation in myopia and glaucoma (Yan and Chaqour, 2013). In glaucoma, pre-treatment of human cribrosa cells with anti-CTGF antibody reduced ECM production such as fibronectin and fibrillin-1 (Wallace et al., 2013)

In summary, CTGF is a central mediator of tissue remodeling and fibrosis. It could interact with protein TGFβ through VWC domain and integrin αvβ3 through CT domain to accumulate ECM and promote fibrosis (Lipson et al., 2012). Moreover, integrins could further promote TGFβ interaction with its receptors, implying VWC domain and CT domain have a synergetic effect in fibrosis (Henderson and Sheppard, 2013). Meanwhile, multiple positive feedback loops of CTGF-TGFβ and CTGF-integrins could enhance the fibrosis progress (Lipson et al., 2012). CTGF could be a promising therapeutic target for fibrosis diseases. CTGF inhibition could enable organs to restore their normal wound healing response and their normal structure and function.

### Inflammatory Diseases

Inflammation is a normal reaction of organs and tissues to protect themselves against a variety of toxic or pathological intrusions (e.g., bacterial infections). The inflammation enables the immune system to efficiently remove the injurious stimuli and initiate the healing process (Chen et al., 2018). In contrast, uncontrolled or sustained inflammation is the underlying cause of many inflammatory diseases such as rheumatoid arthritis (RA) and osteoarthritis (OA). The most common functions of CTGF in inflammatory diseases are to promote the recruitment of immune cells, the production of cytokines, and angiogenesis.

Rheumatoid arthritis is a chronic inflammatory disorder and characterized by leukocyte infiltration, neovascularization, articular cartilage destruction, and synovial membrane inflammation associated with pain and loss of joint function (Kular et al., 2011). Previous studies found that the expression level of CTGF was significantly increased in the fibroblasts and synovial fluid of RA patients compared with the healthy (Wang et al., 2012; Yang et al., 2017). The contribution of CTGF to the pathogenesis of RA comes from the interaction of CTGF domains with its binding proteins. CTGF can enhance pathologic proliferation of T cells and production of interleukin 17 (IL-17) in mouse models through CT domain (Nozawa et al., 2013; Rodrigues-Diez et al., 2013). Apart from pro-inflammatory function, CTGF also causes articular damage by increasing osteoclastogenesis (Nozawa et al., 2009). CTGF could enhance osteoclastic function through the activation of integrin αVβ3 mediated pathways such as ERK1/2 (Nozawa et al., 2009). Multiple CTGF antibodies have shown the ability to neutralize osteoclastogenesis and formation of tubular networks, which indicates that CTGF might serve as a potential therapeutic agent in the treatment of RA (Miyashita et al., 2016).

Osteoarthritis is the most prevalent joint disease and a common cause of joint pain, functional loss, and disability (Robinson et al., 2016). The pathogenesis of OA involves the degradation of cartilage, hypertrophy, and ectopic growth of bony structures in the joints, and the inflammatory cells in the surrounding tissues (Itoh et al., 2013). CTGF has been found to be the most abundantly expressed growth factor in chondrocytes of human patients with severe OA (Zhang et al., 2002). CTGF is usually up-regulated in synovial fluid of OA that stimulates the production of inflammatory cytokines, such as IL-6, which is induced by CTGF-αvβ5-JNK pathways (Liu et al., 2012; Tu et al., 2019). *In vivo*, deletion of CTGF increased the thickness of the articular cartilage and protected mice from OA (Tang et al., 2018). However, administration of recombinant CTGF into defective articular cartilage could regenerate the cartilage in the OA model (Nishida et al., 2004). IGFBP and CT domains have been involved in chondrogenesis (Takigawa, 2013), which might contribute to the protective role of CTGF in OA.

Alzheimer’s disease (AD) is characterized by the deposition of amyloid-β peptide (Aβ) which triggers pro-inflammatory pathways and secrete inflammatory cytokines, and this inflammatory response can become toxic and harmful to neuronal cells (Frost et al., 2019). Elevated CTGF expression is observed in brain neurons and astrocytes from AD patients, and its expression level positively correlates with the progression of clinical dementia (Ueberham et al., 2003). CTGF could facilitate the production of cytokines and chemokines by astrocytes and enhance the recruitment of immune cells such as mononuclear cells, leading to locally augmented immune response (Lu et al., 2019). CTGF also plays important roles in other brain diseases, such as Parkinson’s disease, brain injury, glioblastoma, and cerebral infarction (Mann et al., 2017). However, the underlying mechanisms of CTGF in brain disorders remains unclear.

In inflammatory disorders, CTGF exerts pro-inflammatory functions mostly via interaction with integrin αvβ3 and αvβ5, in which CT domain might be responsible for this function.

## Current Therapeutic Strategy Targeting CTGF

Connective tissue growth factor is considered as a therapeutic target to combat cancer, fibrosis and other related disorders in a variety of organs, and tissues. There are many approaches such as antibodies, synthetic peptides, small interfering RNAs (siRNAs), and antisense oligonucleotides (ASOs), targeting CTGF to exert therapeutic effect (Table 4).

**TABLE 4:** Summary of drugs targeting CTGF.

Drug name	Indication	Development stage	Molecule type	Mechanism
FG-3019	IPF	Phase III	Antibody	Targeting VWC domain (Sgalla et al., 2020)
FG-3019	LAPC	Phase III	Antibody	Targeting VWC domain (Sgalla et al., 2020)
FG-3019	DMD	Phase II	Antibody	Targeting VWC domain (Sgalla et al., 2020)
FG-3019	COVID-19	Phase II	Antibody	Targeting VWC domain (Sgalla et al., 2020)
FG-3149	DCM	Pre-clinical	Antibody	Targeting VWC domain (Koshman et al., 2015)
Unnamed	Pulmonary fibrosis	Pre-clinical	Antibody	Targeting CT domain (Wang X. et al., 2011)
Unnamed	Liver fibrosis	Pre-clinical	Antibody	Targeting TSP1 domain (Tong and Brigstock, 2006)
Unnamed	Systemic sclerosis	Pre-clinical	Antibody	Targeting VWC domain (Ikawa et al., 2008)
BLR-100/BLR-200	PDAC	Pre-clinical	Peptide	Synthetic peptides derived from an endogenous inhibitor (CCN3) of CTGF (Resovi et al., 2020)
RXI-109	Hypertrophic scar/Retinal Scar	Phase II/Phase II	siRNA	Silencing *CTGF* (Barefoot et al., 2018)
OLX-101	Hypertrophic scar	Phase I	siRNA	Silencing *CTGF* (Nikam and Gore, 2018)
OLX-201	Pulmonary fibrosis	Pre-clinical	siRNA	Silencing *CTGF* (Nikam and Gore, 2018)
EXC-001	Hypertrophic scar	Phase II	Antisense oligonucleotide	Silencing *CTGF* (Jensen et al., 2018)

*IPF, idiopathic pulmonary fibrosis; LAPC, locally advanced unresectable pancreatic cancer; DMD: Duchenne muscular dystrophy; COVID-19, coronavirus disease 2019; DCM, dilated cardiomyopathy; PDAC, pancreatic ductal adenocarcinoma.*

### Antibodies and Peptides

Humanized monoclonal antibodies are particularly well suited as blocking agents for CTGF activities and thus hold promise as potential therapeutics. Currently, multiple antibodies targeting different domains of CTGF have been studied. FG-3019 (pamrevlumab), the most widely studied antibody, is a fully human recombinant DNA-derived CTGF-targeted monoclonal antibody (Brenner et al., 2016). FG-3019 targets VWC domain of CTGF and directly against the sequence (Cys142–Gly157) that is not conserved between CCN family members (Wang Q. et al., 2011). FG-3019 could reduce fibrosis in the liver, pancreas, lung, and skeletal muscles (Gonzalez et al., 2018) and block the progression of tumors including mesothelioma, acute lymphoblastic leukemia, ovarian cancer, melanoma and pancreatic cancer in animal studies (Leask, 2020a; Sgalla et al., 2020). FG-3019 has been granted Orphan Drug Designation by the U.S. Food and Drug Administration in several diseases such as IPF and DMD (Leask, 2020b). FG-3019 researches have been in phase 3 clinical trial for the treatment of IPF and locally advanced unresectable pancreatic cancer (LAPC), and in phase 2 clinical trial for the treatment of DMD and acute COVID-19 disease, respectively (Barbe et al., 2020; Herbelet et al., 2020; Sgalla et al., 2020). The proportion of IPF patients with disease progression is significantly lower in the FG-3019 group than in the placebo group (10.0 vs. 31.4%), and FG-3019 demonstrates a safety profile similar to that of placebo (Richeldi et al., 2020). There have been other antibodies in the pre-clinical stage such as FG-3149 and three self-developed antibodies which target VWC, CT, TSP1, and VWC domain of CTGF, respectively, and have shown anti-fibrosis effects (Tong and Brigstock, 2006; Ikawa et al., 2008; Wang X. et al., 2011; Koshman et al., 2015).

It has been reported that CTGF could be inhibited by other CCN family members such as CCN3, and CCN3 could be a potential anti-fibrotic treatment (Peidl et al., 2019). Although the exact mechanism of CTGF inhibition by CCN3 is unclear, two modified synthetic peptides derived from CCN3 (BLR-100 and BLR-200) have shown a promising inhibitory effect on CTGF expression and are evaluated in pre-clinical studies (Resovi et al., 2020). BLR-100 and BLR-200 could reduce tumor angiogenesis, fibrosis, and necrosis in pancreatic ductal adenocarcinoma (PDAC) model, which alters the tumor microenvironment and increases the tumor response to chemotherapy (Resovi et al., 2020).

### siRNAs and ASOs

The other strategies, siRNAs and ASOs, which have a high specificity to the target gene, have also been used in CTGF-targeted therapies. RXI-109 is a new class of stable, CTGF-targeted, self-delivering siRNA (Byrne et al., 2013; Martinez et al., 2015). Intradermal injection of the RXI-109 results in robust, dose-dependent, long-lasting reduction of CTGF in a rodent model of dermal wound healing. Silencing of *CTGF* also impacts both fibrotic markers, myofibroblast differentiation, and collagen deposition (Libertine et al., 2014). A phase 2 clinical study was conducted with RXI-109 to evaluate its impact on the reduction of hypertrophic scar formation after scar revision surgery. The study successfully meets the primary effectiveness objective with statistically significant outcomes for improved visual appearance for RXI-109 treated scar over control. RXI-109 also meets the secondary objective as it is shown to be safe and well tolerated (Barefoot et al., 2018). Another two siRNA drugs, OLX-101 aim at hypertrophic scar is currently under phase I clinical trial. OLX-201 aims at IPF is being evaluated in pre-clinical studies (Nikam and Gore, 2018).

An antisense oligonucleotide (EXC-001) has been developed to inhibit CTGF production and reduce CTGF-driven collagen deposition and scar formation (Jensen et al., 2018). The mechanism of EXC-001 action is to bind to *CTGF* mRNA and inhibit the expression of CTGF protein. In a randomized, double-blind, placebo controlled study, significant reductions in scar severity were observed following treatment with EXC-001 (Jensen et al., 2018). EXC-001 was well tolerated, with no serious adverse effects and no changes in laboratory parameters considered related to the study drug (Jensen et al., 2018).

Current therapeutic strategies targeting CTGF mentioned above have shown moderate to promising results in different diseases. However, there still have some concerns for these strategies. Firstly, anti-CTGF monoclonal antibodies are likely to be rapidly cleared and therefore need to be administered at higher doses and/or more frequently (Brenner et al., 2016). Secondly, the higher rate of infection was observed in FG-3019 group compared to the placebo group (respiratory tract infection: 30% vs. 21%; Urinary tract infection: 20% vs. 8%) (Richeldi et al., 2020), which might suggest that anti-CTGF antibodies have a negative effect on the immune system. Thirdly, anti-CTGF antibodies may exert negative effects on skeletal development since treatment with CTGF antibodies inhibited chondrogenesis *in vitro* (Arnott et al., 2011). In terms of siRNAs and ASOs, there are some limitations including: (i) low specificity that can lead to toxicities, (ii) poor cellular delivery as a result of difficulties in biological membrane crossing (Chery, 2016; Setten et al., 2019). Although they have shown promising results in anti-scarring, more investigations are needed to test the therapeutic promise in tumors and severe fibrotic disorders such as IPF.

## The Perspective for the Drug Discovery of the Next Generation of CTGF Inhibitors

### Structural Elements for CTGF-Targeted Therapies

Through the interaction of CTGF domains with a variety of proteins, the deregulation of CTGF expression or activity contributes to the pathobiology of various diseases including cancers, fibrotic disorders, and inflammatory diseases. In cancers and fibrotic disorders, both CT domain and VWC domain contribute to the development of diseases, and these two domains may have a synergic role. For example, tumor migration can be activated by the EMT process from the binding between VWC domain with TGFβ (Wendt et al., 2010; Zhu et al., 2015), and the angiogenesis from the binding between of CT domain with integrin αvβ3 (Wang L. H. et al., 2017). Moreover, both interactions of CTGF(VWC)-TGFβ and CTGF(CT)-αvβ3 contribute to the ECM accumulation in fibrotic disorders (Gao and Brigstock, 2004; Lipson et al., 2012). Multiple domains should be taken into consideration for the maximizing the anti-fibrosis or anti-tumor effects. The targeting sequence needs to be carefully selected since the interaction of CT domain and LRP6 contributes to skeletal development (Kanaan et al., 2006). CTGF shares an identity of 30–50% in amino acid sequence with other CCN family proteins which have demonstrated critical biological functions (Schild and Trueb, 2004). The drugs targeting CTGF should not interfere with the function of other CCN family members, therefore, the less conserved motif in CTGF is a better choice for drug discovery.

### New Technology for Drug Discovery Targeting CTGF

Although antibodies are now established as a key therapeutic modality for a range of diseases, their limited stability, complicated *in vivo* production, and typically undefined cross-reactivity are challenges to overcome (Voskuil, 2014). Aptamers are short (20–70 bases) single stranded oligonucleotides that bind to their targets through 3D conformational complementarities with high affinity and specificity (Yu et al., 2016). The aptamer-based drug ‘Macugen’ was approved by the Food and Drug Administration in 2004 for the treatment of neovascular age-related macular degeneration and a series of aptamer-based drugs are in clinical pipelines (Zhang et al., 2020). Aptamers are considered to be strong chemical rivals of antibodies due to their inherent advantages over antibodies. Compared to antibodies: (i) aptamers can be produced using cell-free chemical synthesis and are therefore less expensive to manufacture, (ii) aptamers exhibit extremely low variability between batches and have better controlled post-production modification, (iii) aptamers are minimally immunogenic, (iv) aptamers are more stable at room temperature and longer shelf life, and (v) aptamers are small in size and could bind to regions which are inaccessible to antibodies (Kaur et al., 2018). Moreover, aptamers could specifically target a specific protein from family proteins with highly similar structures (Yu et al., 2016).

In summary, CTGF has four domains that interact with a variety of proteins such as growth factors, integrins, and matrix proteins. These interactions affect multiple signaling pathways and make CTGF a key regulator and potential therapeutic target in fibrotic, inflammatory diseases, and cancers. A variety of CTGF antagonistic strategies have been developed in numerous experimental systems to reverse these diseases. Novel CTGF-targeted therapeutic strategies, such as aptamer-based CTGF-targeted strategies, are desired for the treatment of these diseases.

## Author Contributions

ZC did the literature research and wrote the manuscript. NZ, HC, and YY helped in polishing the manuscript. Z-KZ, GZ, and B-TZ revised and approved the manuscript. All the authors contributed to the article and approved the submitted version.

## Conflict of Interest

The authors declare that the research was conducted in the absence of any commercial or financial relationships that could be construed as a potential conflict of interest.

## Supplementary Material

Supplementary Figure 1 Fifty models created for each domain of Ctgf and full length Ctgf, the model presenting the lowest value of Dope (Discrete Optimized Protein Energy) were chosen as the best structure. **(A–E)** Model number 48 (Dope value: −4030) for Igfbp domain; model number 23 (Dope value: 4472) for Vwc domain; model number 2 (Dope value: −1842) for Tsp1 domain; model number 18 (Dope value: −3555) for Ct domain; model number 12 (Dope value: −17078) for full length Ctgf.

Supplementary Table 1 The contacted residues (distance less than 8 Angstroms).
