# Supplementary material for: Connective Tissue Growth Factor: From Molecular Understandings to Drug Discovery
Source: Front Cell Dev Biol. 2020 Oct 29;8:593269. doi: 10.3389/fcell.2020.593269 (PMC7658337; doi:10.3389/fcell.2020.593269)
Supplement: Supplementary Figure 1 — Fifty models created for each domain of Ctgf and full length Ctgf, the model presenting the lowest value of Dope (Discrete Optimized Protein Energy) were chosen as the best structure. (A–E) Model number 48 (Dope value: −4030) for Igfbp domain; model number 23 (Dope value: 4472) for Vwc domain; model number 2 (Dope value: −1842) for Tsp1 domain; model number 18 (Dope value: −3555) for Ct domain; model number 12 (Dope value: −17078) for full length Ctgf. [file Table_1.docx]

**Supplementary Table SI.** The contacted residues (distance less than 8 Angstroms)

| **Correlated residues** | **Distance** | **Correlated residues** | **Distance** | **Correlated residues** | **Distance** | **Correlated residues** | **Distance** |
| --- | --- | --- | --- | --- | --- | --- | --- |
| VWC:ASP140-TSP1:ASN198 | 2.95 | VWC:LEU136-TSP1:ALA197 | 6.778 | VWC:SER118-CT:PHE319 | 5.694 | VWC:MET134-TSP1:TRP206 | 7.582 |
| VWC:CYS161-TSP1:ALA197 | 3.211 | VWC:GLY132-TSP1:GLU205 | 6.806 | VWC:PRO151-TSP1:SER227 | 5.738 | VWC:GLU162-TSP1:CYS199 | 7.584 |
| VWC:CYS137-TSP1:ALA197 | 3.4 | VWC:ALA130-TSP1:CYS209 | 6.813 | VWC:MET139-TSP1:ASN198 | 5.749 | VWC:SER119-CT:LYS315 | 7.587 |
| VWC:CYS137-TSP1:CYS199 | 3.642 | VWC:LYS159-TSP1:ALA197 | 6.832 | VWC:GLU162-TSP1:ASN198 | 5.767 | VWC:CYS160-TSP1:ALA197 | 7.601 |
| VWC:SER118-CT:MET318 | 3.952 | IGFBP:CYS95-VWC:GLY113 | 6.847 | VWC:SER119-CT:MET317 | 5.83 | VWC:GLU163-TSP1:ALA197 | 7.605 |
| VWC:ARG152-TSP1:SER227 | 4.005 | VWC:ARG153-TSP1:SER227 | 6.89 | IGFBP:CYS95-VWC:LEU127 | 5.837 | VWC:GLY132-TSP1:THR204 | 7.609 |
| VWC:LYS121-TSP1:CYS199 | 4.086 | VWC:TYR122-TSP1:CYS199 | 6.979 | VWC:ALA130-TSP1:CYS213 | 5.853 | VWC:LEU127-TSP1:CYS209 | 7.67 |
| VWC:CYS161-TSP1:ASN198 | 4.842 | VWC:GLY106-CT:MET318 | 7.049 | VWC:PRO135-TSP1:GLN202 | 5.89 | VWC:GLY129-TSP1:THR212 | 7.679 |
| VWC:CYS137-TSP1:ASN198 | 4.958 | VWC:LYS155-CT:ASN316 | 7.059 | VWC:ASP140-TSP1:ALA197 | 5.93 | VWC:GLU162-TSP1:LEU200 | 7.692 |
| VWC:PRO135-TSP1:THR204 | 5.04 | VWC:LYS121-TSP1:LEU200 | 7.06 | VWC:MET139-CT:GLY272 | 6.012 | VWC:GLY132-TSP1:CYS209 | 7.699 |
| VWC:SER138-CT:CYS273 | 5.084 | VWC:SER119-CT:MET313 | 7.07 | VWC:CYS133-TSP1:THR204 | 6.173 | VWC:PRO151-TSP1:GLU231 | 7.701 |
| VWC:PRO135-TSP1:CYS199 | 5.101 | VWC:ARG153-TSP1:LEU200 | 7.14 | IGFBP:THR96-VWC:GLY113 | 6.184 | VWC:LEU143-TSP1:VAL201 | 7.708 |
| VWC:ASP140-CT:SER271 | 5.176 | VWC:VAL141-CT:GLY272 | 7.147 | VWC:ASP140-CT:GLY272 | 6.208 | VWC:CYS137-TSP1:LEU200 | 7.722 |
| VWC:CYS160-CT:ASN316 | 5.18 | TSP1:ALA197-CT:SER271 | 7.184 | VWC:LEU136-TSP1:CYS199 | 6.212 | TSP1:ASN198-CT:GLY272 | 7.747 |
| VWC:CYS161-TSP1:CYS199 | 5.276 | VWC:SER119-CT:PHE319 | 7.187 | VWC:VAL141-CT:SER271 | 6.273 | VWC:GLY106-CT:PHE319 | 7.76 |
| VWC:PHE150-TSP1:CYS228 | 5.306 | VWC:VAL141-TSP1:ASN198 | 7.187 | VWC:SER119-CT:ASN316 | 6.316 | TSP1:ASN198-CT:SER271 | 7.76 |
| VWC:MET134-TSP1:THR204 | 5.372 | VWC:TYR122-TSP1:GLN202 | 7.227 | VWC:SER138-TSP1:ALA197 | 6.406 | VWC:THR108-CT:PHE319 | 7.807 |
| IGFBP:ASP99-VWC:SER112 | 5.431 | VWC:PRO149-TSP1:CYS228 | 7.228 | VWC:GLU163-CT:SER271 | 6.413 | VWC:ALA130-TSP1:GLY214 | 7.825 |
| VWC:GLU163-TSP1:ASN198 | 5.474 | IGFBP:LYS98-VWC:ASP128 | 7.228 | VWC:CYS120-CT:ASN316 | 6.419 | VWC:GLU162-CT:ASN316 | 7.863 |
| TSP1:ALA197-CT:LEU270 | 5.488 | IGFBP:ASP99-VWC:ASP128 | 7.232 | VWC:SER138-TSP1:ASN198 | 6.521 | VWC:CYS120-TSP1:CYS199 | 7.867 |
| VWC:PHE150-TSP1:SER227 | 5.624 | VWC:ARG153-TSP1:GLU231 | 7.287 | VWC:PRO157-CT:PRO330 | 6.603 | VWC:MET139-TSP1:VAL201 | 7.876 |
| VWC:ALA130-TSP1:THR212 | 5.637 | VWC:CYS160-TSP1:CYS199 | 7.29 | VWC:ARG153-TSP1:ASN198 | 6.661 | VWC:GLY132-TSP1:CYS213 | 7.89 |
| VWC:SER138-CT:GLY272 | 5.68 | TSP1:ALA197-CT:GLY272 | 7.324 | VWC:LYS121-CT:ASN316 | 6.689 | VWC:PRO135-TSP1:VAL201 | 7.898 |
| VWC:SER119-CT:MET318 | 5.69 | VWC:PRO135-TSP1:LEU200 | 7.45 | VWC:TRP164-TSP1:ASN198 | 6.695 | VWC:SER118-CT:MET317 | 7.921 |


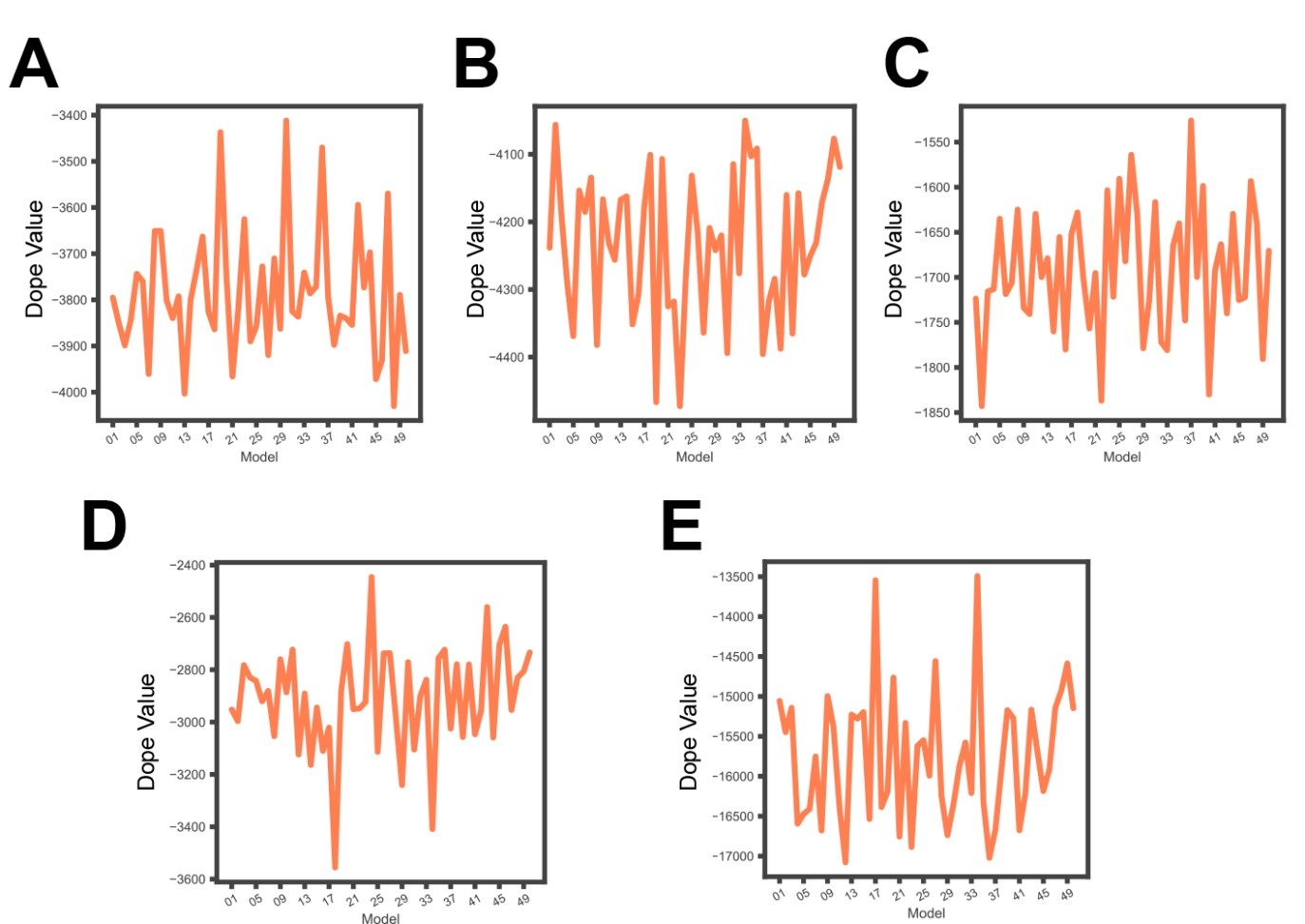


**Supplementary Figure S1.** Fifty models created for each domain of Ctgf and full length Ctgf, the model presenting the lowest value of Dope (Discrete Optimized Protein Energy) were chosen as the best structure. (**A-E**) Model number 48 (Dope value:−4030) for Igfbp domain; model number 23 (Dope value: 4472) for Vwc domain; model number 2 (Dope value:−1842) for Tsp1 domain; model number 18 (Dope value:−3555) for Ct domain; model number 12 (Dope value:−17078) for full length Ctgf.
